# Influence of olive cake dietary supplementation on fecal microbiota of dairy cows

**DOI:** 10.3389/fmicb.2023.1137452

**Published:** 2023-05-03

**Authors:** Nunziatina Russo, Viviana Floridia, Enrico D’Alessandro, Vincenzo Lopreiato, Alessandra Pino, Vincenzo Chiofalo, Cinzia Caggia, Luigi Liotta, Cinzia Lucia Randazzo

**Affiliations:** ^1^Department of Agriculture, Food and Environment, University of Catania, Catania, Italy; ^2^ProBioEtna SRL, Spin-Off of University of Catania, Catania, Italy; ^3^Animal Production Unit, Department of Veterinary Sciences, University of Messina, Messina, Italy; ^4^CERNUT, Interdepartmental Research Centre in Nutraceuticals and Health Products, University of Catania, Catania, Italy; ^5^Consortium Research of Meat and Agribusiness Chain, Messina, Italy

**Keywords:** olive by-products, stool samples, metagenomics, microbiota, enzymatic pathways

## Abstract

Olive by-products represent a valuable low-price feed supplement for animal nutrition. In the present study, the effect of the dietary destoned olive cake supplementation, on both composition and dynamics of the fecal bacterial biota of cow, was assessed by Illumina MiSeq analysis of the 16S rRNA gene. In addition, metabolic pathways were predicted by using the PICRUSt2 bioinformatic tool. Eighteen lactating cows, according to the body condition score, the days from calving, and the daily milk production were homogeneously allocated into two groups, control or experimental, and subjected to different dietary treatments. In detail, the experimental diet contained, along with the components of the control one, 8% of destoned olive cake. Metagenomics data revealed significant differences in abundance rather than in richness between the two groups. Results showed that *Bacteroidota* and *Firmicutes* were identified as the dominant phyla, accounting for over 90% of the total bacterial population. The *Desulfobacterota* phylum, able to reduce sulfur compounds, was detected only in fecal samples of cows allocated to the experimental diet whereas the *Elusimicrobia* phylum, a common endosymbiont or ectosymbiont of various flagellated protists, was detected only in cows subjected to the control diet. In addition, both *Oscillospiraceae* and *Ruminococcaceae* families were mainly found in the experimental group whereas fecal samples of control cows showed the presence of *Rikenellaceae* and *Bacteroidaceae* families, usually associated with the high roughage or low concentrate diet. Based on the PICRUSt2 bioinformatic tool, pathways related to carbohydrate, fatty acid, lipid, and amino acids biosynthesis were mainly up regulated in the experimental group. On the contrary, in the control group, the metabolic pathways detected with the highest occurrence were associated with amino acids biosynthesis and degradation, aromatic compounds degradation, nucleosides and nucleotides biosynthesis. Hence, the present study confirms that the destoned olive cake is a valuable feed supplement able to modulate the fecal microbiota of cows. Further studies will be conducted in order to deepen the inter-relationships between the GIT microbiota and the host.

## 1. Introduction

Recently, the use of agro-industrial by-products as feed supplements in animal nutrition represents an interesting and successfully adopted strategy to reduce feeding costs and satisfy the nutritional needs of livestock (Chiofalo B. et al., 2020; Chiofalo V. et al., 2020; El Otmani et al., 2021; Bionda et al., 2022). In this context, the use of by-products of the olive oil industry, such as destoned olive cake, became widespread as a feed supplement since they are rich in nutraceutical molecules with antioxidant and antimicrobial features, including polyphenols (flavonoids, anthocyans, cyanidins, and phenolic acids), tyrosol, hydroxytyrosol, and oleuropein (Mannelli et al., 2018; Foti et al., 2022). Recent evidence suggests that the use of olive by-products did not negatively impact nutrients utilization, microbiota composition, and rumen fermentation variables, such as pH, ammonia and volatile fatty acids (VFA) concentrations (Ruiz et al., 2004; Estaún et al., 2014; Pallara et al., 2014; Tzamaloukas et al., 2021). Differently by influencing the ruminal fermentation and the rumen pH, the increase of the content of linoleic acid, in both milk and meat, as well as change in the aromatic and microbiological profiles of milk are reported (Chiofalo V. et al., 2020; Liotta et al., 2020; Foti et al., 2021; Tzamaloukas et al., 2021; Rabee et al., 2022). In fact, the high polyphenol content could modulate the rumen microbiota biodiversity and, consequently, affect the rumen metabolism, decreasing dietary protein degradation and fatty acid biohydrogenation by means of targeting specific groups of microorganisms (Mannelli et al., 2018; Biondi et al., 2019; Milani et al., 2020).

To date, modern sequencing technologies, based on culture-independent methods, represent the most powerful tools available for elucidating the diversity of animal microbiomes, positioning the microbial ecology of cattle for its renaissance (Hagey et al., 2019; Vaccalluzzo et al., 2020). In addition, the significant advances in sequence data analysis allow deep insights into this ecosystem (Hagey et al., 2019). The vast majority of available data, related to the microbial community identification in cattle, is focused on the rumen microbiota and less attention was paid to the study of fecal samples (Dowd et al., 2008; Shanks et al., 2011; Tang et al., 2017). Nevertheless, fecal sampling represents a non-invasive way suitable to link changes in bacterial abundance and diversity, along with related functional traits, to bovine rumen microbiome, with a high level of reproducibility and repeatability (Mott et al., 2022). In fact, the deepening of the fecal microbiota can provide valuable insights into the effect of feeding strategies on the nutritional status and wellbeing of livestock along with helping in manure management mitigating the environmental impact of pollution (Hagey et al., 2019). Recently published data, using 16S rRNA gene sequencing-based approaches, suggest that by studying the fecal microbiota of cattle is possible to elucidate the effects of a variety of feeds (Dowd et al., 2008; Callaway et al., 2010; Rice et al., 2012; Li et al., 2017; Kotz et al., 2021).

According to that, the main goal of the present study was to investigate the effect of the dietary destoned olive cake supplementation on fecal microbiota of cows using 16S rRNA gene amplicon sequencing. In addition, metabolic pathways, at KEGG level 3, were predicted by using the PICRUSt2 bioinformatic tools.

## 2. Materials and methods

### 2.1. Animal welfare

The Ethical Committee of the Department of Veterinary Science of the University of Messina approved all procedures (code 041/2020), conducted according to the European guidelines for the care and use of animals in research (Directive 2010/63/EU).

### 2.2. Animal management and diet

**FIGURE 1:**
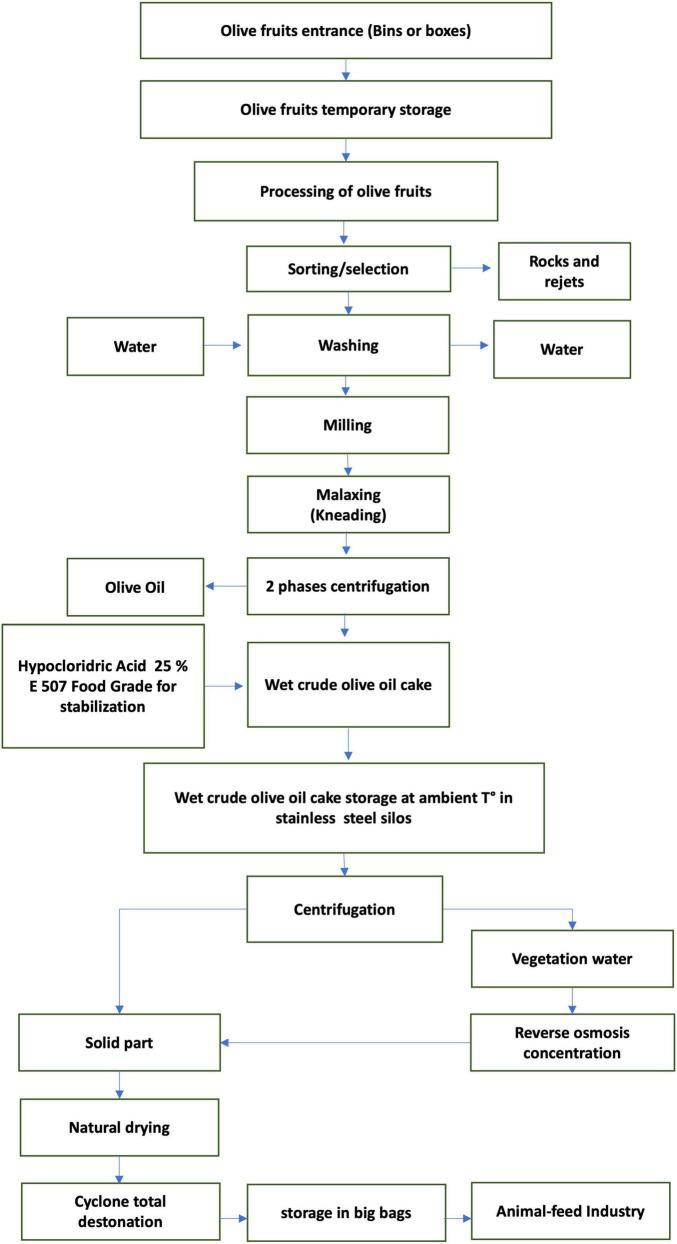
Flowchart of the destoned olive cake production.

This experiment was conducted for 100 days (from February to June 2022) in a commercial dairy farm located 520 m above sea level in the province of Ragusa (Sicily, Italy). A total of 18 lactating cows, homogenous for the Body Condition Score (3 ± 0.5), distance from calving (90–120 days), and daily milk production (25 ± 3 kg/day) were randomly allocated in two groups, control (CTRL; 9 cows) or experimental (TRT; 9 cows). In detail, all cows were fed as total mixed ration (TMR) once daily at 0700 h where diet was composed by concentrate and meadow hay. The CTRL group received a concentrate without any olive cake integration, whereas the TRT group received a concentrate integrated with the enriched olive cake at the inclusion of 8%. The chemical composition of concentrates is reported in Table 1. The enriched olive cake, used as supplement, was obtained by mechanical pressing of olives carried out through a two-stage process, applied to produce extra virgin olive oil, by adding about 5% of a concentrate of vegetation waters, subsequently pitted by centrifugation and dried in the open air. The chemical composition (on DM basis) of the olive cake used in the present experiment was as follow: 95.6% of dry matter, 8.6% of crude protein, 30.3% of ether extract, 49.4% of neutral detergent fiber, 39.4% of acid detergent fiber, 23.1% of acid detergent lignin, 4.1% of ash, 1.5% of starch, and 9.360 mg/kg of polyphenols. A flowchart of olive cake production is reported in Figure 1.

**TABLE 1:** Nutritional characteristics of concentrates used in the experiment.

Diet	CTRL	TRT
**Chemical composition, g/kg of dray matter (DM)**		
Moisture	109	107
Starch	407	407
Crude protein	194	196
Ether extract	45.8	51.1
Non-fiber carbohydrates	465	440
Crude fiber	60.0	72.0
Acid detergent fiber	78.2	105
Ash	64.1	70.2
**Calculated nutrient composition**		
NE_L_, milk UFL/kg of DM	1.09	1.07

NEL, net energy lactation. Milk production efficiency was calculated based on the net energy system, where one milk forage unit (UFL) of energy is defined as the net energy content of 1 kg of standard barley for milk production, equivalent to 1700 kcal.

### 2.3. Fecal samples collection and DNA extraction

Fecal samples were collected, from each cow, 100 days after the start of the control or experimental diet administration. Samples were aseptically collected from the rectal ampoule by using sterile gloves then placed in sterile containers and transferred under refrigerated conditions to the Laboratory of Microbiology of the Department of Agricultural Food and Environment (University of Catania, Italy) and immediately frozen at −80°C until analysis. Total genomic DNA was extracted using the commercial QIAamp^®^ DNA Stool Mini Kit (QIAgen, Hilden, Germany) following the manufacturer instructions with a slight modification consisting of a repeated bead beating (RBB) pre-treatment step (Randazzo et al., 2015). In detail, after melted on ice, 0.25 g of feces was weighted under sterile conditions and transferred into a 2 ml screw-cap tube containing four glass-beads (2.7 mm, Biospec Products, Inc., USA) and 0.5 g of zirconia beads (0.1 mm, Biospec Products, Inc., USA). After the addition of 1 ml of lysis buffer (50 mM Tris–HCl, 500 mM NaCl, 50 mM EDTA, 4% w/v sodium dodecyl sulfate), the sample was subjected to mechanical cell lysis by performing three rounds of bead-beating with Precellys 24 Tissue Homogenizer (Bertin Technologies, Montigny-le-Bretonneux, France). Treatment time was 3 min for each bead-beating round and samples were cool on ice in between. After the RBB treatment the samples were centrifuged at + 4°C for 5 min at full speed and the collected supernatant was used for the DNA extraction following the kit manufacturer’s instructions. DNA concentration was evaluated using the fluorimeter Qubit 4.0 (Invitrogen, Carlsbad, CA, United States) before storing at −20°C until use.

### 2.4. 16S rRNA gene library construction and sequencing

The fecal microbiome composition was determined by 16S rRNA gene sequencing as previously described (Milani et al., 2013; Vaccalluzzo et al., 2022). Briefly, the V3 region of the 16S rRNA gene was amplified using PCR to build qualified libraries, which were subjected to MiSeq (Illumina) sequencing at the facilities of GenProbio Srl.^1^ The obtained 16S rRNA raw data were deposited at NCBI Sequence Read Archive (SRA)^2^ under accession code PRJNA909483.

### 2.5. Bioinformatic analysis

The raw reads were processed using Quantitative Insights Into Microbial Ecology (QIIME2) version 2022.2 (Bolyen et al., 2019). The sequences were quality filtered, trimmed, and denoised using Divisive Amplicon Denoising Algorithms 2 (DADA2). The high-quality sequences were then used to construct the amplicon sequence variant (ASV) feature table. Taxonomic classification was made through the SILVA reference database (v138) (Robeson et al., 2021) with a percentage of identity of 75, 87, and 95% for phylum, family, and genus levels, respectively (Henderson et al., 2019). ASVs with relative abundance lower than 0.1% were grouped as “others.”

### 2.6. Alpha and beta diversity, differential analysis, and enzymatic prediction

The determination of diversity and differences in the abundance of the fecal bacterial community was performed using RStudio software (version 4.1.2). The *phyloseq* packing of R (McMurdie and Holmes, 2013) was used for alpha and beta diversity. The alpha diversity, based on genus level, was evaluated considering three measures: observed richness, Chao1 index, and Shannon index. The box plot of alpha diversity was generated using the *ggplot2* package (Wickham, 2016). Beta diversity, based on genus level, was evaluated with the Bray-Curtis distance and plotted with the Principal Coordinate Analysis (PCoA). Differential analysis, based on genus level, was performed using the *DESeq2* package in R (Love et al., 2014) and the differences between CTRL and TRT groups were evaluated.

The Phylogenetic Investigation of Communities by Reconstruction of Unobserved States (PICRUSt2) v2.5.0 (Douglas et al., 2020) was used to predict the functional abundances at KEGG level 3 (i.e., Environmental Information Processing) based on 16S rRNA gene sequencing data obtained during the denoise step by QIIME2. Statistical analysis of taxonomic and functional profiles (STAMP) (v2.1.3) (Parks et al., 2014) was used to illustrate, based on the PICRUSt2 outputs, the difference in the predicting microbial functions associated with the diet.

### 2.7. Statistical analysis

The alpha diversity, according to observed richness, Chao1 and Shannon indices, between CTRL and TRT groups was compared with one-way non-parametric Wilcoxon test. Beta diversity, according to Bray Curtis distance, was evaluated through permutational multivariate analysis of variance (PERMANOVA) with 999 permutations with the *vegan* package.

Differential abundance (DA) was considered with a false discovery rate (FDR) cut-off of 0.05 and a fold-change (FC) higher than 1.5 or lower than −1.5 (i.e., |log2FC| > 0.59). The difference in the prediction of microbial functions associated with the diet (CTRL vs. TRT) was detected with Welch’s *t*-test with a confidence interval of 95% and the data were corrected with Benjamini–Hochberg FDR. Significance was determined at *P* ≤ 0.05.

## 3. Results

### 3.1. Taxonomy classification

**FIGURE 2:**
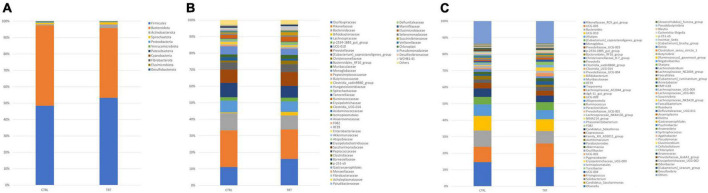
Relative abundance of phyla **(A)**, families **(B)**, and genera **(C)** detected in fecal samples from cows subjected to control (CTRL) and experimental (TRT) diet.

After trimming, denoising, chimera-removal and merging with QIIME2, the obtained high-quality sequences were used for the taxonomic classification. Sample metadata, denoising statistics, taxonomy classification and the relative abundances for each taxonomic level are reported in Supplementary Data 1. As reported in Table 2, the total ASVs were assigned to 10 phyla, 51 families and 78 genera in CTRL group, whereas 10 phyla, 44 families and 78 genera were detected in TRT group. The relative abundance of bacteria, detected in both CTRL and TRT groups, at phylum, family, and genus levels is shown in Figures 2A–C. In detail, *Firmicutes*, *Bacteroidota*, *Actinobacteriota*, *Spirochaetota*, *Proteobacteria*, *Verrucomicrobiota*, *Patescibacteria*, *Cyanobacteria*, and *Fibrobacterota* phyla were detected in both CTRL and TRT groups with relative abundance higher than 0.1%. Although *Firmicutes* and *Bacteroidota* were the most abundant phyla in both CTRL and TRT groups, the *Firmicutes* phylum showed higher relative abundance in TRT samples compared with CTRL. Moreover, the *Desulfobacterota* and *Elusimicrobia* phyla were not detected in CTRL and TRT groups, respectively (Figure 2A).

**TABLE 2:** Overall microbiota composition of fecal samples collected from cows subjected to control (CTRL) and experimental (TRT) diet.

	% Identity	CTRL	TRT
Phylum	75%	10	10
Family	87%	51	44
Genus	95%	78	78

**FIGURE 3:**
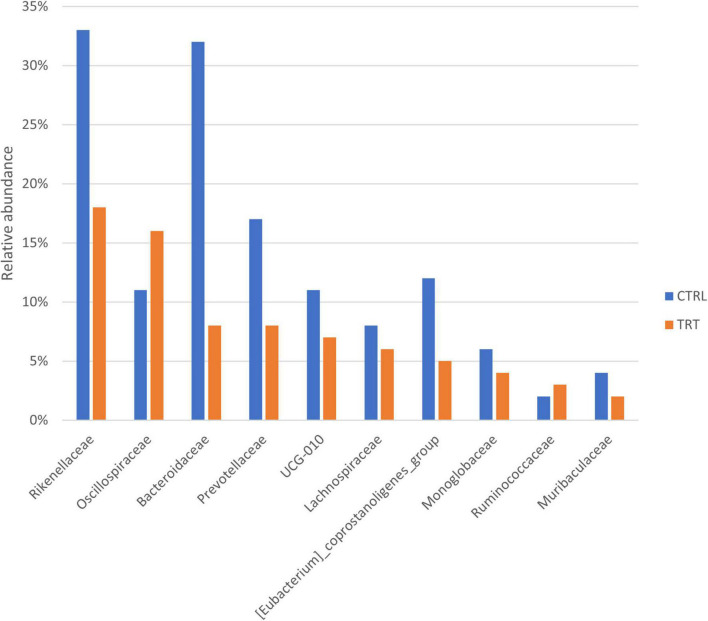
Top 10 most abundant families detected in control (CTRL) and experimental (TRT) groups.

Overall, at family level, the ASVs of CTRL group were classified into 51 families, whereas the ASVs of TRT group were classified into 44 families (Figure 2B). In fact, compared with CTRL group, the *Peptococcaceae*, *Defluviitaleaceae*, *Elusimicrobiaceae*, *Succinivibrionaceae*, *Veillonellaceae*, *Pseudomonadaceae*, and *WCHB1-*41 families where not detected in TRT group. As reported in Figure 3, showing the ten most abundant families, *Oscillospiraceae* and *Ruminococcaceae* were dominant in in TRT group, whereas *Rikenellaceae* and *Bacteroidaceae* were most abundant in CTRL group.

**FIGURE 4:**
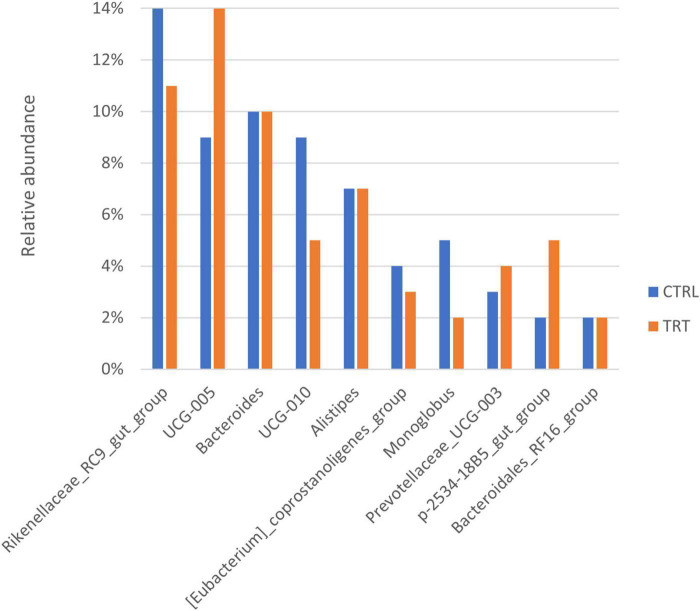
Top 10 most abundant genera detected in control (CTRL) and experimental (TRT) groups.

At genus level, 78 genera were overall identified in both CTRL and TRT groups (Figure 2C) and the most abundant are reported in Figure 4. In particular, *Rikenellaceae_RC9_gut_group*, *UCG-010 and Monoglobus* showed higher relative abundance in CTRL group, while *UCG-005*, *Prevotellaceae_UCG-003* and *p-2534-18B5_gut_group* were prevalent in TRT group (Figure 4).

Moreover, *Roseburia*, *Prevotellaceae_Ga6A1_g -roup*, *Cellulosilyticum*, *Gastranaerophilales*, *[Eubacterium]_siraeum_group*, *Lachnospiraceae_UCG-009*, *Dielma*, *Odoribacter*, *Anaeroplasma*, *Faecalibacterium*, *Chloroplast and Desulfovibrio* were not found in CTRL group whereas *Negativibacillus*, *Acinetobacter*, *Agathobacter*, *Erysipelotrichaceae_UCG-002*, *Defluviitaleaceae_UCG-011*, *Anaerovorax*, *Elusimicrobium*, *Anaerovibrio*, *Succinivibrio*, *Syntrophococcus*, *Psychrobacter*, *and Pseudomonas* were not found in TRT group (Figure 2C).

### 3.2. Alpha and beta diversity, differential analysis, and enzymatic prediction

Observed richness, Chao1, and Shannon indices are plotted in Supplementary Figures 1A–C. Based on the Wilcoxon test, no difference in richness was detected between CTRL and TRT groups.

**FIGURE 5:**
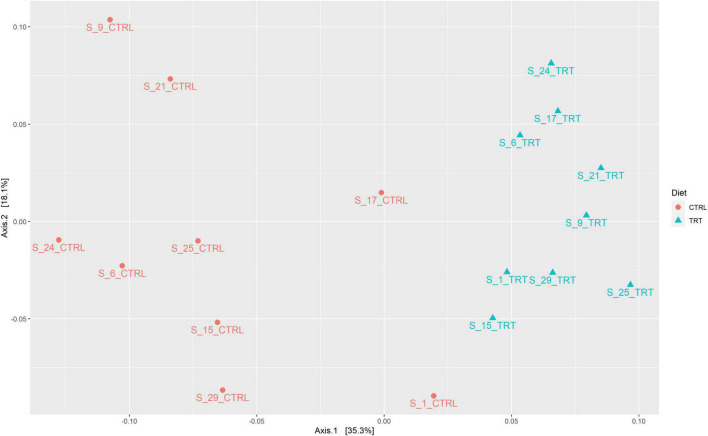
Principal coordinate analysis (PCoA) depicting the distribution of samples according to Bray—Curtis distance.

The beta diversity (Figure 5), based on the Bray-Curtis distance method, allowed to group the analyzed samples in relation to the dietary treatment (CTRL or TRT). Based on PERMANOVA results, the diet significantly affected the fecal microbial communities (*p* < 0.001).

The model used in the differential analysis allowed to detect the genera, occurring in both CTRL and TRT groups, with significant differences in terms of percentage of occurrence (Table 3).

**TABLE 3:** Different taxa identified by comparing the fecal microbiota of cows allocated to control (CTRL) and experimental (TRT) groups.

Phyla	Family	Genus	Log2 fold change	*p*-value	*p*-value-adj
*Firmicutes*	*Christensenellaceae*	*Christensenellaceae_R-7_group*	0.92098*	0.001	0.006
	*RF39*	*RF39*	1.72107*	0.001	0.001
	*Clostridia_UCG-014*	*Clostridia_UCG-014*	1.12519*	0.001	0.001
	*Lachnospiraceae*	*Acetitomaculum*	−1.95689**	0.001	0.005
*Bacteroidota*	*Bacteroidales_RF16_group*	*Bacteroidales_RF16_group*	−0.69586**	0.001	0.001

*More prevalent in TRT group. **Less prevalent in TRT group.

**FIGURE 6:**
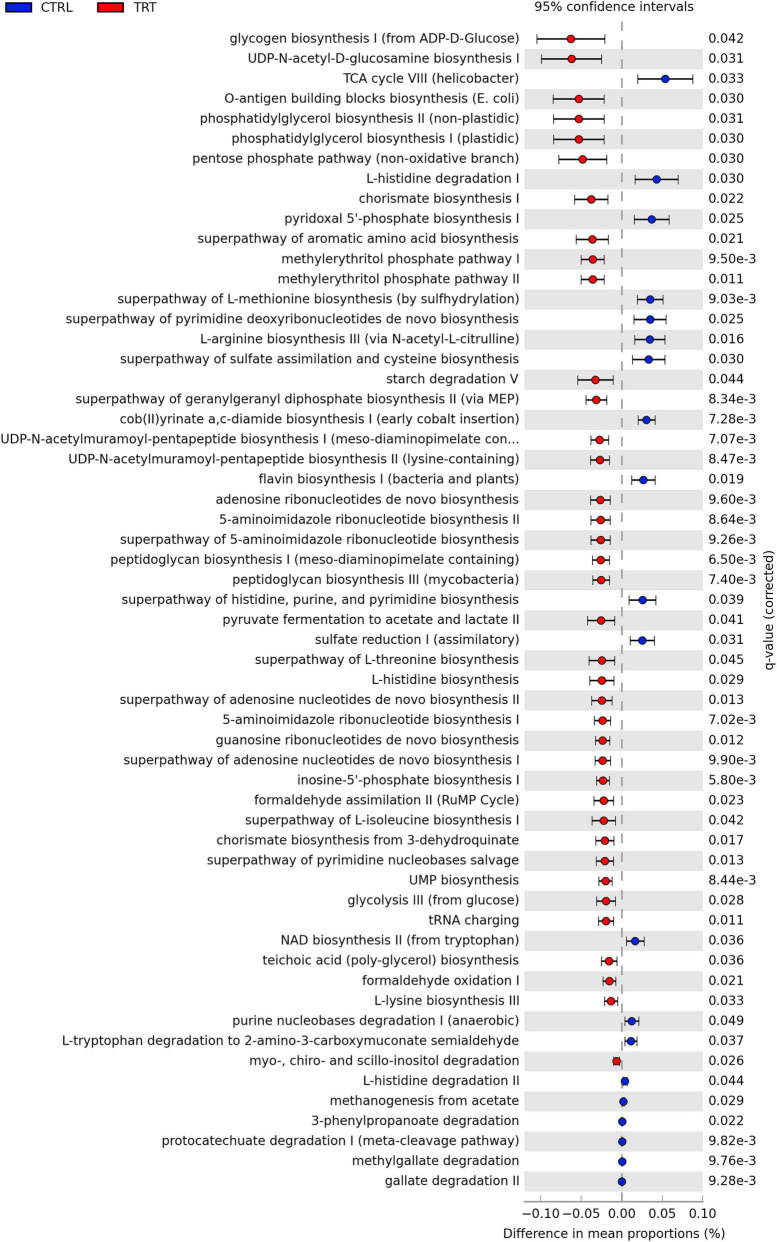
Differentially metabolic pathways between control (CTRL) and experimental (TRT) groups.

Overall, 366 pathways were detected based on KEGG prediction (Supplementary Data 2) and 58 of them, with different abundance between CTRL and TRT groups, are shown in Figure 6. In detail, in TRT group, the metabolic pathways mainly identified were involved in the biosynthesis of carbohydrates (Glycogen biosynthesis I, UDP-*N*-acetyl-D-glucosamine biosynthesis I, O-antigen building blocks biosynthesis), fatty acids and lipids (Phosphatidylglycerol biosynthesis I and II), and amino acids (Superpathway of aromatic amino acid biosynthesis, Superpathway of L-threonine biosynthesis, L-histidine biosynthesis, Superpathway of L-isoleucine biosynthesis I and L-lysine biosynthesis III). In the CTRL group, the identified pathways were mainly involved in amino acids degradation (L-histidine degradation I and III) and biosynthesis (L-arginine biosynthesis III and Superpathway of L-methionine biosynthesis) as well as in aromatic compounds degradation and nucleosides and nucleotides biosynthesis (Figure 6).

## 4. Discussion

The development of alternative feedstuffs and the use of by-products, as feed supplements, represent a challenge for animal nutrition researchers to boost farm livestock wellbeing as well as increase both the production and quality of animal-derived products. The diet becomes a key factor contributing to changes in the composition of the gastrointestinal tract (GIT) microbiota (Zhang et al., 2021; Welch et al., 2022). According to that, the present study, by applying a metagenomic approach, aimed to deepen the effect of the dietary destoned olive cake supplementation on both composition and dynamics of the fecal bacterial biota of cow as well as on the KEGGs functional profile.

Consistent with previously reported data, our study confirmed that polyphenols rich diet is able to modulate the microbial community affecting, in turn, the fecal microbiota composition (Mao et al., 2013; Plaizier et al., 2017; Hagey et al., 2019). In fact, although no significant difference in richness was detected, beta diversity allowed to discriminate the analyzed samples based on the diet regime. Metagenomics approaches revealed that the fecal microbiota of cattle is dominated by *Firmicutes* and *Bacteroidetes* phyla, commonly recognized as health-promoting (Henderson et al., 2015; Mannelli et al., 2019; Conte et al., 2022; Welch et al., 2022). More in depth, *Firmicutes* are involved in the degradation of oligosaccharide, fiber, and starch, helping the host intestinal tract in the absorption of energy from food. In addition, members of the *Firmicutes* phylum are able to produce volatile fatty acids such as butyrate, which is linked with gut health (Kim et al., 2011). Similarly, members of the *Bacteroidetes* phylum have many functions in the gut, including degradation of carbohydrates, such as complex plant cell walls, as well as production of butyrate, a significant player in energy metabolism in the rumen (Thomas et al., 2011; Miguel et al., 2019). It is well known that the *Firmicutes* phylum acts by increasing the nutrients availability whereas the *Bacteroidetes* one is energetically less favorable to the host (Xu et al., 2021).

According to previous studies, suggesting that dietary changes significantly affect the *Firmicutes:Bacteroidota* ratio (Mao et al., 2013; Plaizier et al., 2017, 2018), our data revealed increased abundance of *Firmicutes* and decreased occurrence of *Bacteroidota* in fecal samples of cows subjected to destoned olive cake supplementation. In addition, the *Desulfobacterota* phylum was detected only in fecal samples of treated cows. Noteworthy, the aforementioned phylum includes microorganisms able to reduce sulfur compounds via the sulfite reductase pathway, followed by butyrate degradation, playing a significant role in energy metabolism (Miguel et al., 2019). Moreover, *Elusimicrobia*, a recently defined animal-associated phylum, occurring as endosymbiont or ectosymbiont of various flagellated protists (Méheust et al., 2020), was detected only in cows subjected to control diet. The presence of both *Oscillospiraceae* and *Ruminococcaceae* families mainly in experimental group can be associate to the diet regime. In fact, as recently reported by Yang et al. (2021), polyphenols can increase the abundance of the families mentioned above which are able to ferment complex plant carbohydrates and to produce short chain fatty acids competing with activation of energy metabolism. Conversely, fecal samples of control cows showed the presence of *Rikenellaceae* and *Bacteroidaceae* families, usually associated with the high roughage or low concentrate diet (Mpanza et al., 2022), playing a key role in carbohydrates degradation and in the production of VFAs, including succinate, acetate and propionate (Wang et al., 2020). Consistent with metagenetic data, in the present study, most of the predicted metabolic pathways are involved in biosynthetic processes. Among these, pathways related to carbohydrate, fatty acid, lipid and amino acids biosynthesis were mainly present in experimental group than in control one. This finding is in accordance with the observed dominance of both *Bacteroidota* and *Firmicutes* phyla in fecal microbiota. In fact, it is well known that *Firmicutes* are involved in the degradation of complex polysaccharides, with subsequent synthesis of VFA, whereas *Bacteroidetes* mainly degrades carbohydrates, fats, and proteins (Jami et al., 2013; Yildirim et al., 2021). On the contrary, in control group, the metabolic pathways detected with highest occurrence were associated to amino acids biosynthesis and degradation, aromatic compounds degradation, nucleosides and nucleotides biosynthesis. It is well known that bacteria require the synthesis and/or acquisition of purines and pyrimidines, which form the basis of nucleotides, to survive, even having strong links with the virulence factors of opportunistic and bacterial pathogens (Goncheva et al., 2022). In fact, in most bacteria, the nucleotides are synthesized *de novo* and the products are used in many cell functions, including DNA replication, energy storage, and as signaling molecules (Goncheva et al., 2022). Our results confirm that the feeding regime significantly affect the composition and dynamics of the fecal microbiota as well as the microbial metabolism. Further studies will be conducted in order to in depth investigate the molecular functions of microbiota by using insightful methods, such as metabolomics and metatranscriptomics.

## 5. Conclusion

The present study confirms that, among olive by-products, destoned olive cake is a valuable feed supplement for cow’s nutrition. The destoned olive cake supplementation was able to modulate the fecal microbiota determining the increase of *Firmicutes* phylum, associated to growing nutrients availability, and the reduction of *Bacteroidetes*, energetically less favorable to the host. The prediction of metabolic pathways revealed a significant effect of the regime diet on carbohydrate, fatty acid, lipid and amino acids biosynthesis. Further studies will be conducted in order to deepen the inter-relationships between the GIT microbiota and the host.

## Data availability statement

The datasets presented in this study can be found in online repositories. The names of the repository/repositories and accession number(s) can be found in the article/Supplementary material.

## Ethics statement

The study was reviewed and approved by the Ethical Committee of the Department of Veterinary Science of the University of Messina (code 041/2020). All procedures were conducted according to the European guidelines for the care and use of animals in research (Directive 2010/63/EU). Written informed consent was obtained from the owners for the participation of their animals in this study.

## Author contributions

LL, CR, and VL: conceptualization. AP, VC, and CR: methodology. VF and AP: software. VL, AP, LL, and CR: validation. NR and VF: formal analysis and writing—original draft preparation. VL and LL: investigation. VF, ED’A, AP, and LL: data curation. VL, AP, CC, LL, and CR: writing—review and editing. LL and CR: visualization and supervision. LL: project administration and funding acquisition. All authors have read and agreed to the published version of the manuscript.

## Footnotes

1
www.genprobio.com

2
https://www.ncbi.nlm.nih.gov/sra

## Supplementary material

Supplementary Figure 1 Alpha diversity based on **(A)** Observed, **(B)** Chao1, and **(C)** Shannon index. Statistical analysis provides the difference of richness at genus level in the two groups (CTRL vs. TRT).

Supplementary Data 1 16S rRNA raw data.

Supplementary Data 2 Abundance of each pathway detected by prediction analysis.
