# Supplementary figures and images for: Influence of olive cake dietary supplementation on fecal microbiota of dairy cows
Source: Front Microbiol. 2023 May 3;14:1137452. doi: 10.3389/fmicb.2023.1137452 (PMC10188969; doi:10.3389/fmicb.2023.1137452)

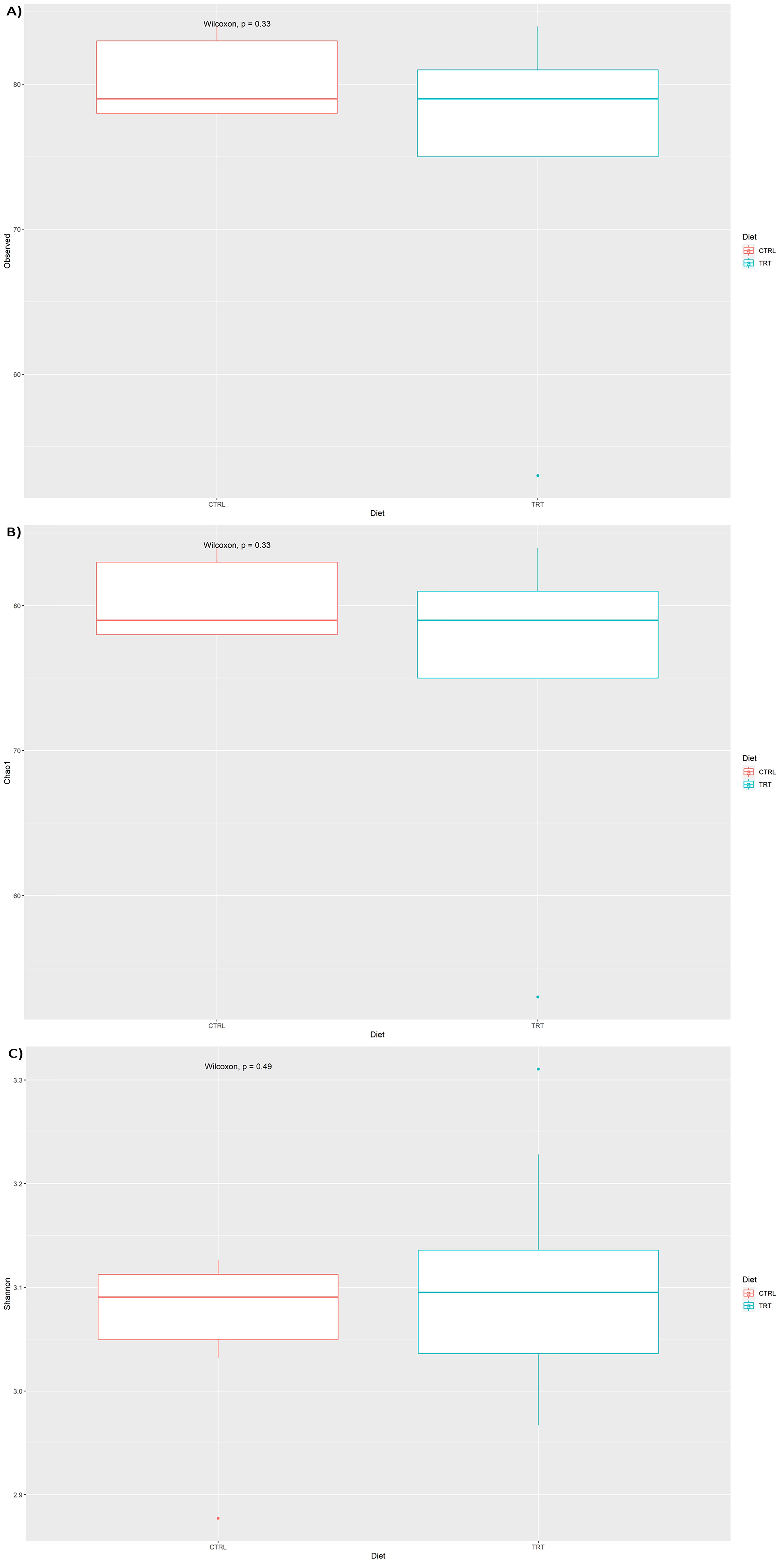

Supplement: Supplementary Figure 1 — Alpha diversity based on (A) Observed, (B) Chao1, and (C) Shannon index. Statistical analysis provides the difference of richness at genus level in the two groups (CTRL vs. TRT). [file Image_1.TIFF]
